# Extensive Preferential Pathway Ablation for the Elimination of Premature Ventricular Contractions Arising from the Right Ventricular Outflow Tract

**DOI:** 10.1016/s0972-6292(16)30610-6

**Published:** 2013-03-07

**Authors:** Norishige Morita, Takayuki Iida, Ueno Akira, Yoshinori Kobayashi

**Affiliations:** 1 Division of Cardiology, Department of Medicine, Tokai University Hachioji Hospital, Tokyo; 2 Division of Cardiology, Department of Medicine, Nippon Medical School, Tokyo, Japan

**Keywords:** Preferential pathway, Pre-potential, Right Ventricular outflow tract, Premature ventricular contraction, Ablation

## Abstract

A 76 y/o women presented with 2 different types of premature ventricular contractions (VPCs 1 and 2) arising from the right ventricular outflow tract (RVOT). Catheter ablation (CA) eliminated PVC1 at the earliest activation site (EAS), but thereafter another PVC morphology (PVC3) appeared. Small potentials preceding the local potential were broadly exhibited from the RVOT's supero-anterior region to the EAS during PVC3. Point CA targeting such pre-potentials failed. Transverse-linear CA with a line connecting sites with such pre-potentials eliminated both PVCs 3 and 2. In cases with broadly spreading preferential pathways, extensive CA might be needed to eliminate the PVCs.

## Introduction

Most premature ventricular contractions (PVCs) arising from the right ventricular outflow tract (RVOT) can be cured by the radiofrequency catheter ablation (RFCA). The earliest activation site (EAS) during the PVCs has been known to be the best target site for RFCA eliminating those PVCs . Although such an approach targeting the EAS with RFCA has been known to eliminate the PVCs, there have been some cases in which the RFCA of the EAS during the PVCs could not result in the elimination of the PVCs even though pace mapping from the EAS exhibited an excellent match to the QRS morphology during the spontaneous PVCs. In general RFCA applications for the EAS are appropriate for curing the PVCs when the PVCs arise from an identical site as the EAS. If the PVC arises from a site away from the EAS, whether the origin is located in the end/mid, or epicardial myocardium, such an approach targeting the EAS might fail to eliminate the PVC since it is possible that the EAS reflects the activation of an endocardial exit site to which the excitation from the PVC origin might preferentially propagate [1,2]. Here we present a case with RVOT-PVCs which could not be eliminated by point RFCA applications at the EAS, but could be eliminated by a linear RFCA with a line intersecting the area where small potentials were recorded preceding the local ventricular activation.

## Case Report

**Figure 1:**
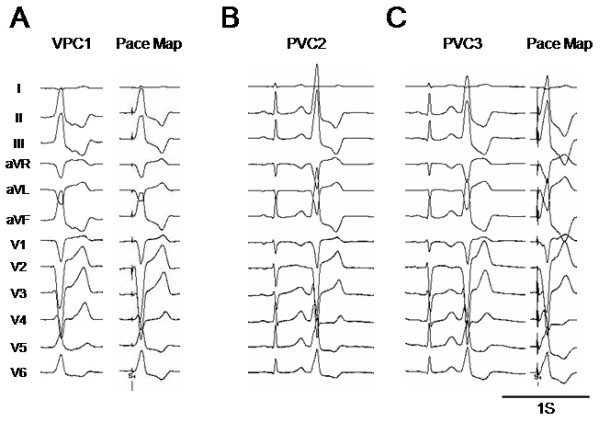
12-lead ECGs demonstrating two types of premature ventricular contractions (PVCs). (A) The morphology of PVC1, which was the main morphology documented, was a left bundle branch block pattern with a superior axis (left panel). The 12-lead ECGs during pace mapping from the earliest activation site (EAS) during PVC1 (right panel). (B) The 12-lead ECGs of PVC2 were similar to those of PVC1, but the amplitude of the R wave in the inferior leads was greater than that of PVC1 and the transitional zone of the R/S wave differed. (C) Newly appearing PVC (PVC3) after the RFCA of PVC1 (left panel). The 12-lead ECGs during pace mapping at the EAS during PVC3 (right panel).

**Figure 2:**
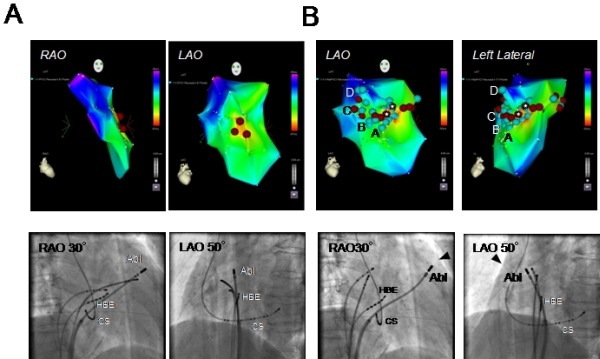
(A) Activation map during PVC1 (upper panels) and the ablation catheter position in the fluoroscopic views (lower panels). The earliest activation site (EAS) is shown in red and the latest in purple. (B) Activation map during PVC3 (upper panels) and the ablation catheter position in the fluoroscopic views (lower panels). The ablation catheter was positioned at Point A corresponding to the site indicated on Figure. 4. The presumable borderline between the right ventricular outflow tract and pulmonic artery is indicated by the arrowhead. The asterisks indicate the initial ablation points targeting the sites exhibiting the pre-potential during PVC3. The light-blue circles indicate the sites where the pre-potentials were recorded and the red ones the sites where the ablation was performed. Abl=ablation catheter, CS=recording catheter for coronary sinus electrograms, HBE=recording catheter for His-bundle electrograms, LAO=left anterior oblique projection RAO=right anterior oblique projection. Refer to the text for the details.

**Figure 3:**
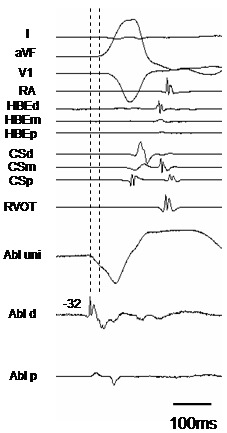
The intracardiac electrograms during PVC1. The local potential recorded from the ablation catheter which was located at the EAS preceded the QRS onset by 32ms. Shown from the top to bottom are the body surface ECG leads I, aVF, and V1 and the intracardiac signals from the right atrium (RA), proximal, mid-, and distal His-bundle electrogram recording sites (HBEp, HBEm, and HBEd, respectively), proximal, mid-, and distal coronary sinus (CSd, CSm, and CSp, respectively), right ventricular outflow tract (RVOT), unipolar electrogram recorded from the distal electrode of the ablation catheter (Abl uni), and the distal and proximal pair of electrodes of the ablation catheter located at the EAS in the right ventricular outflow tract (Abl d and Abl p, respectively). Refer to the text for the details.

**Figure 4:**
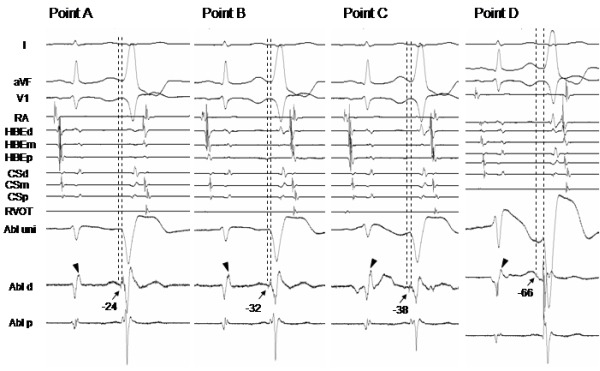
(A)The intracardiac electrograms recorded from Points A to D during PVC3, which correspond to each site shown in Fig 2B. The numerical numbers shown on each figure indicate the difference in time between the emergence of the pre-potentials recorded on the local electrograms and QRS onset during PVC3.The abbreviations are the same as in Fig. 3. Refer to the text for the details.

A 76 y/o women experienced frequent episodes of palpitations and consulted our hospital. The 12-lead ECG exhibited normal sinus rhythm with a PQ interval and QRS width of 100 and 66 ms, respectively. The QT and QTc intervals were 520 and 425ms, respectively. Two types of PVCs were documented on the 12-lead ECG, and the morphology of one PVC (PVC1) exhibited a left bundle branch block with an inferior axis, and the other (PVC2) was similar to PVC1 but its QRS morphology was slight different (PVC2) (Figure 1). Routine check-ups including a transthoracic echocardiogram revealed no structural abnormalities. Her Holter monitoring revealed 21,564 PVC beats over 24 hrs which coincided in time with her palpitations. She was admitted to our hospital and underwent an electrophysiologic study (EPS). In the baseline study, the atrio-His and His-to-ventricular intervals during sinus rhythm were 135 ms and 42ms, respectively. Based on the morphologies of those two types of PVCs, the sites from which the PVCs arose were thought to be located in the right ventricular outflow tract (RVOT) [3]. The earliest activation site (EAS) during the PVCs was determined using an electroanatomical mapping system (CARTO XP, Biosense Webster, Diamond Bar, CA). Since PVC1 was more frequently documented than PVC2 during the EPS, mapping of PVC1 was performed first to try and cure PVC1. The EAS during PVC1 was located in the anteroseptal region of the RVOT (Figure 2A), where the local excitation preceded the QRS onset by 32ms and a QS complex was recorded by the mapping catheter in the unipolar electrogram (Figure 3). The pace map from the EAS exhibited an excellent match to the QRS morphology during spontaneous PVC1 (Figure 1A). RFCA was applied at the EAS and to sites within close proximity to the EAS determined by CARTO during PVC1, and are indicated by red tags in Figure 2A. Those RFCA applications eliminated PVC1, but another type of PVC (PVC3) with a different morphology from that of PVCs 1 and 2 appeared with a similar frequency to PVC1 (Figure 1C). Therefore a new electro-anatomical map was created to define the EAS during PVC3 after the initial RFCA applications targeting PVC1. The mapping revealed that the EAS during PVC3, where the local excitation preceded the QRS onset by 24ms, was located in the septal region of the RVOT and around 15mm more posterior than the EAS during PVC1 (Figure 2B). The pace map performed from the EAS during PVC3 also revealed a good match (Figure 1C). Again the RFCA applications to the EAS and the area around the EAS during PVC3 as indicated by the asterisks in Figure 2B however, could not eliminate PVC3. Careful mapping around the EAS during PVC3 revealed a small potential preceding the local ventricular potential when PVC3 appeared, and is indicated by the arrows in Figure 4. The region where such potentials could be recorded is indicated by the light blue tags in Figure 2B. Such potentials could be seen during the late phase of the QRS complex and might be superimposed by the local ventricular potential during sinus rhythm, as indicated by the arrowheads in Figure 4. The sites where such pre-potentials could be recorded covered a relatively large area of the RVOT, and were observed mainly in the superior region, both anteriorly and posteriorly, to the EAS, and in particular beneath the pulmonic valve as indicated by the arrowheads in Figure 2B. Although this was not determined with ventriculography, the position of the pulmonic valve could be assumed from the cardiac silhouette. The spatio-temporal relationship between the antecedent timing of the pre-potential and the emergence of the onset of the QRS, and the location where the pre-potentials could be recorded is shown in Figure 2B and 4. The pre-potentials appeared earlier in timing to the QRS onset as the recording site became further away from the EAS. To assess the electrophysiologic relationship between such pre-potentials and the emergence of PVC3, pacing from the sites demonstrating such potentials was performed. The selective capture of such pre-potentials without local ventricular capture by pacing however, was impossible. A few applications of RFCA targeting the sites exhibiting such potentials, as indicated by the asterisks in Figure 2B, were delivered to see if they had any influence on suppressing the incidence of PVC3, but the RFCA did not have any influence on either the frequency or morphology of PVC3. Based on the results of the failure to eliminate PVC3 by RFCA at a few points, we presumed that the origin of PVC3 was located at a site some distance away from the EAS and that the excitation was propagating from the PVC origin through several preferential pathways, resulting in a single phenotypic presentation of the PVC3 QRS morphology even though the course of the conduction differed. The strategy for the RFCA applications was changed to create RFCA regions that intersected an arbitrary line connecting points between the sites where the pre-potentials could be recorded. The linear but actually stepped linear RFCA applications between the posterior and anterior septum in the RVOT, as indicated by the red tags in Figure 2B, resulted in the complete elimination of PVC3. In the process of delivering those RFCA applications for PVC3, the frequency of the occurrence of PVC2 decreased. Since PVC2 never appeared again after the final RFCA application targeting PVC3, the session was completed. During 6 months of follow-up, no further occurrences of PVCs 1-3 have been documented.

## Discussion

To eliminate RVOT-PVCs with RFCA, the precise localization of their origin reflected as the EAS during the PVC has been thought to lead to a successful RFCA. In addition, a pace mapping procedure is usually performed to ensure whether pacing from the EAS can elicit the same QRS morphologies in the 12-lead ECG as those during spontaneous PVCs, thus confirming that the EAS is a proper site for RFCA. An estimation based on the results obtained from pace mapping however, has been known to be limited because of the capture of a large area of the myocardium around the EAS or a rate-dependent alternation in the conduction during pacing. Another complementary procedure for searching for the appropriate site for RFCA is known to be searching for a site exhibiting a QS complex pattern recorded in the local unipolar electrogram. This method also has the limitation of the surrounding area of the EAS exhibiting a QS morphology pattern in an area. Because of these limitations on the determination of the proper RFCA site, the complete elimination of the PVCs often requires several RFCA applications not only at the EAS, but also in the surrounding area in close proximity to the EAS. Therefore it seems that the EAS sometimes might move to a site close to the EAS which was initially identified, when VPCs still emerge after the initial RFCA applications. Further, when the EAS moves to a site > 10mm away from the initially identified EAS and the PVC morphology changes after several RFCA applications to the EAS and the area within close proximity to the EAS, this may imply that the substrate of the PVC origin involves a relatively large area with a few exist sites located in the area neighboring the EAS or another PVC becomes unmasked from a different origin. In our case, although the distance between the EAS during PVCs 1 and 3 was more than 10mm, the frequency of the incidence of PVC3 was almost same as that of PVC1. Therefore it could be possible that the origin of PVCs 1 and 3 was a common substrate containing a relatively large area and the direction of the conduction from the PVC origin became altered after the initial RFCA application for PVC1, resulting in the change in the QRS morphology from PVC 1 to 3. Even if the origin of both PVCs 1 and 3 were composed of such a substrate, the failure to eliminate PVC3 by a further RFCA application to the EAS as well as the area in close proximity to the EAS during PVC3, led us to consider that the PVC origin might be located distant from the EAS since pace mapping from the EAS during PVC3 exhibited a good match. Careful mapping around the endocardial EAS during PVC3 exhibited small potentials preceding the local ventricular activation. Cases in which the PVC was expected to arise from the RVOT or pulmonic artery (PA) have been reported to exhibit small pre-potentials preceding the local excitation in the PA during the PVC, as was the case in our current report [1,2,4]. In addition, an alteration in the QRS morphology of the PVC and increased amplitude of the R wave in the inferior leads was seen after the RFCA applications at the EAS during the PVC in cases with PVCs arising from the PA, and that was a similar finding to that in our case [5]. In such cases, the RFCA application to the EAS failed to eliminate the PVC, but that to the site where the pre-potentials were recorded lead to the elimination of the PVC. The EAS during PVC3 in our case was thought to be located in the RVOT based on the catheter position on fluoroscopy, but this was not actually determined with ventriculography. A part of the RFCA sites superior to the EAS during PVC3 exhibiting small pre-potentials might have been located in the PA (Figure 2B). The sites that exhibited pre-potentials, were spread out over a relatively large area, anterior and posterior to the EAS located in the RVOT septal region. When the RFCA application at the EAS and its vicinity during the RVOT-PVCs fails to eliminate the PVCs or changes the morphology of the PVC, careful mapping in the neighboring area of the RVOT should be made to search for small potentials preceding the local excitation during PVCs. Further, when some point RFCA applications to sites where pre-potentials are recorded can not eliminate the PVCs, a linear RFCA to intersect a line connecting the sites exhibiting pre-potentials may be a useful procedure. In our case, PVC2 disappeared after this procedure to cure PVC3. The reason why PVC2 disappeared could not actually be addressed, but one consideration might be that the RFCA of some preferential pathways arising from the origin of PVC3, which might also have been connected to the exit site during PVC2, resulted in the elimination of PVC2. It may be concluded that the careful mapping of the neighborhood of the EAS to find pre-potentials during RVOT-PVCs might aid in leading to the successful elimination of the PVCs when PVCs still appear after several RFCA applications to the EAS and its vicinity.
